# Diet, Physical Activity and Depression: Does Gastrointestinal Health Help Explain the Relationship Between Lifestyle Factors and Depression?

**DOI:** 10.1111/nbu.12734

**Published:** 2025-03-04

**Authors:** Deili Sinimeri, Caroline Childs, Dennis Golm

**Affiliations:** ^1^ School of Psychology, Faculty of Environmental and Life Sciences, Centre for Innovation in Mental Health University of Southampton Southampton UK; ^2^ School of Human Development and Health, Faculty of Medicine University of Southampton Southampton UK

**Keywords:** depression, diet, exercise, functional gastrointestinal disorders

## Abstract

Lifestyle factors such as diet and physical activity are involved in the development and maintenance of depression, but the mechanism by which these factors influence mental health remains unclear. The aim of this study was to investigate whether gastrointestinal health helps explain some of the relationship between these lifestyle factors and depression. The study used a cross‐sectional design to compare dietary intake, physical activity and gastrointestinal health in three groups, healthy (*n* = 235), lifetime depression (*n* = 161) and current depression (*n* = 86). Dietary intake was measured by the Fruit And Vegetable VAriety index, N‐3 PUFA Food Frequency Questionnaire and Prebiotic and Probiotic Food Frequency Questionnaire. Analysis of variance, Pearson correlations and Hayes PROCESS macro mediation analysis were used to compare the groups and examine the relationships. Physical activity and gastrointestinal health differed significantly between the groups with no differences in overall fruit and vegetable, omega‐3 and probiotic food intake. Bootstrapped correlations showed that higher fruit and vegetable and omega‐3 intake were associated with lower gastrointestinal symptom and depression scores, but effects were weak. Furthermore, higher occurrence of gastrointestinal symptoms was moderately associated with higher depression scores. Results from a series of exploratory mediation analyses suggested that gastrointestinal symptoms mediated the relationship between lifestyle factors and depression status. These data indicate that the effects of lifestyle factors on depression might partly work through the gastrointestinal system. The findings of this study help further understand the mechanisms between dietary intake and physical activity, and depression and can inform future longitudinal and experimental studies.

## 1 Introduction

Depression is a low mood disorder that affects approximately 280 million people worldwide (Vos et al. 2020), determined by a complex interplay of biological, psychological and social factors (Remes et al. 2021). Lifestyle factors may play a significant role in the development and maintenance of depression (Marx et al. 2021; Ignácio et al. 2019). Many of these factors can be potentially modified, yet they receive little consideration in the contemporary treatment of depression, where medication and psychological intervention remain the first‐line treatments (National Institute for Health and Care Excellence 2022). Though helpful to a large proportion of the population, medical treatment is not effective for all (McIntyre et al. 2014) and comes with troublesome side effects such as weight gain and sexual dysfunction (Kelly et al. 2022). And more than half do not respond to psychological therapies (Cuijpers et al. 2021).

Meta‐analytic evidence across multiple prospective cohort studies has shown a protective role of physical activity (PA) for depression (Guo et al. 2022; Pearce et al. 2022). The meta‐analysis by Pearce and colleagues only included prospective studies and showed that people adhering to either 50% or the full recommendations for marginal metabolic equivalent task hours per week had a reduced depression risk (18% and 25%, respectively). Additionally, PA has potential as a treatment for depression and can show effects comparable to antidepressants. High‐quality evidence from a meta‐meta‐analysis (Rebar et al. 2015) and a recent umbrella review (Singh et al. 2023) of randomised controlled trials reported medium antidepressant effects of PA.

Dietary intake is also important to consider when thinking about depression. As evidenced across a number of randomised controlled trials, the Mediterranean diet is extensively associated with good physical and mental health (Ventriglio et al. 2020). The Mediterranean diet consists of polyphenol‐rich foods including fruits, vegetables, nuts, seeds, legumes and whole grains (Bayes et al. 2020). In a systematic review including 17 experimental studies, higher polyphenol consumption reduced depressive symptoms (Bayes et al. 2020). A systematic review and meta‐analysis reported that vegetable consumption was related to reduced depression risk. The size of the reduction differed by study design with a 25% reduction of depression risk across cross‐sectional and 18% reduced risk across cohort studies (Saghafian et al. 2018).

Consumption of foods rich in omega‐3 fatty acids or polyunsaturated fatty acids (PUFAs) such as fish, nuts and seeds is strongly related to heart and brain health (Dighriri et al. 2022; Shahidi and Ambigaipalan 2018), and increasingly linked to depression. An umbrella review of meta‐analyses across prospective longitudinal studies reported a protective effect of omega‐3 consumption on depression (Xu et al. 2021). RCTs that used EPA supplementation alongside patients' usual antidepressants found significant improvements in depression symptoms in recurrent major depressive disorder (MDD) and bipolar depression (Osher and Belmaker 2009).

There is also emerging evidence for foods with probiotic properties showing benefits for mental health. Probiotics are microorganisms known to improve gut health through molecular mechanisms that involve various bioactive compounds present in fermented foods (Martirosyan and Leem 2019). A large‐scale cross‐sectional study found that the prevalence of depression was significantly lower in individuals who reported higher rates of probiotic food consumption (Kim and Shin 2019). A 2016 systematic review of RCTs of probiotic consumption found mixed results within healthy populations and a significant reduction in depressive symptoms in MDD (Pirbaglou et al. 2016).

The mechanism between physical activity and dietary intake, and depression remains unclear. There is growing evidence for the inflammation theory of depression which suggests that systemic and neuroinflammation play a key role in the development of depression (Berk et al. 2013; Maeng and Hong 2019). Individuals with depression have increased levels of inflammatory markers (Lindqvist et al. 2017; Tolkien et al. 2019). Physical activity may regulate these abnormalities by adjusting inflammatory markers (Lavebratt et al. 2017; Schuch et al. 2014).

Dietary intake is also known to lead to biochemical changes related to the aetiology of depression. In their meta‐analysis of four prospective cohorts and two cross‐sectional studies, Wang et al. (2019) found that individuals with high intake of proinflammatory foods such as processed foods had a 23% higher risk of developing depression. The risk remained similar (25%) when constraining results to prospective cohort studies. Flavonoid‐rich foods such as fruits and vegetables, and fatty fish reduce inflammation markers in animal studies with a lack of studies in humans (Zhu et al. 2018).

There is a growing body of evidence linking depression to gastrointestinal (GI) or gut health (Nikolova, Cleare, et al. 2021; Nikolova, Hall et al. 2021). Gut health refers to the function and balance of many parts of the gastrointestinal tract. Poor gut health is related to reduction in microbial diversity and lack of ‘good bacteria’ (Rinninella et al. 2019). The human gut is colonised by up to 1000 different species, mainly made up of bacteria (Zoetendal et al. 1998). The gut microbiome communicates with the brain via the bidirectional gut–brain axis through the vagus nerve (Hashimoto 2023). Gut microbial composition plays a role in various health‐promoting processes such as regulatory functions of the immune system including the up‐ or downregulation of peripheral and central inflammatory processes. For instance, chronic inflammation of the gut can weaken the intestinal wall (‘leaky gut’) which enables endotoxins produced by gut bacteria and other metabolites to enter the bloodstream. This can compromise the integrity of the blood–brain barrier, thereby promoting neuroinflammation (Kouba et al. 2024) which has been implicated as a mechanism in the development of major depressive disorder (MDD). In line with this, adults with a diagnosis of MDD show a pattern of gut microbiome alterations characterised by a depletion of anti‐inflammatory and increases of proinflammatory species (Nikolova, Hall et al. 2021). Gut microbiota have been shown to differ significantly when comparing individuals with MDD to healthy populations (Knudsen et al. 2021). Furthermore, depression is often comorbid with irritable bowel syndrome, a common condition that affects the digestive system (Simpson et al. 2020).

Changes in microbiota or the gut microbial composition and leaky‐gut syndrome have further been implicated in the development of functional gastrointestinal disorders and symptoms such as abdominal pain, and changes in bowel movement such as constipation and diarrhoea (Wei et al. 2021).

A host of studies has shown that gut microbiome composition can be positively impacted through the administration of probiotics. The term ‘probiotic’ refers to ‘live microorganisms that, when administered in adequate amounts, confer a health benefit on the host’ (Hill et al. 2014; page 2) with the central idea to increase the number of health‐promoting bacteria and thereby decreasing the proportion of health‐deteriorating bacteria. Key benefits associated with probiotic intake include a strengthening of the intestinal wall and anti‐inflammatory effects (Halloran and Underwood 2019).

An umbrella meta‐analysis of 10 meta‐analyses supports the effectiveness of probiotic supplements in the reduction of depressive symptoms in adults (Musazadeh et al. 2022), particularly in addition to antidepressant medication (Nikolova, Cleare, et al. 2021).

Diet and physical activity are associated with changes in the gut microbial composition. Dietary intake is considered one of the main factors related to the composition of the gut microbiota (Wilson et al. 2020). The foods we eat feed the microbes in the gut, and certain foods, particularly those rich in fibre (Ye et al. 2022), are related to development and diversity of health‐promoting microbes. Several studies have shown that physical activity is associated with qualitative and quantitative changes in microbial composition in humans and that athletes have greater microbiota diversity (Gallè et al. 2019).

The current study used self‐report GI health measures as a proxy for gut microbiome alterations to explore its relationship with dietary intake, PA and depression. The aim of this study was to investigate whether GI health helps explain the relationship between the lifestyle factors and depression.

## 2 Methods

Ethical approval was gained from the University of Southampton's School of Psychology Ethics Committee (ERGO ID: 71124). Anonymised data are available for noncommercial use from the University of Southampton's data repository ePrints (https://eprints.soton.ac.uk).

### 2.1 Participants

G Power 3.1 was used to conduct an a priori power analysis, based on a study with similar design investigating a mediating role of catastrophising beliefs between depression and abdominal pain (Lackner et al. 2004). This suggested a minimum of 124 participants per group to detect large effects of *f* ^2^ = 0.80 at an *α* = 0.05 level of statistical significance and 80% power (1−*β*).

A total of 496 adults (aged ≥ 18 years) consented to participate, who had been previously screened for the presence (*n* = 249) or absence (*n* = 247) of a self‐reported diagnosis of depression. The Physical Health Questionnaire‐9 (PHQ‐9; Kroenke, Spitzer, and Williams 2001) was used to determine individuals with current MDD. Participants in the comparison group without a self‐reported MDD diagnosis were excluded if they currently met the criteria for MDD. Participants were further excluded if they had gastrointestinal disorders with hereditary elements, such as Crohn's disease, ulcerative colitis and coeliac disease and food allergies diagnosed by a clinician. Three attention control questions were included at the beginning, mid and end of the data collection to ensure participants' attention was at a high level to ensure reliability of data (Berinsky et al. 2014). Respondents were excluded if two out of three of these questions were answered incorrectly.

Participants with a self‐reported lifetime MDD diagnosis were divided into two groups. This resulted in three groups: healthy controls (HC, ie, without self‐reported or MDD diagnosis, *n* = 235), lifetime depression (LD, ie, with self‐reported depression, but no current MDD, *n* = 161) and current depression (CD, ie, with self‐reported depression with current MDD, *n* = 86). The demographic information and clinical characteristics are presented in Table 1.

**TABLE 1:** Demographic information and clinical characteristics.

	Healthy (HC) (*n* = 235)	Lifetime depression (LD) (*n* = 161)	Current depression (CD) (*n* = 86)
Age range, *M* (SD)	19–80, 41.81 (13.96)	19–67, 39.86 (12.17)	20–59, 37.22 (11.05)
Sex (% female)	60.7%	62.7%	65.1%
Ethnicity	85.5% White, Black, 2.1%, 7.7% Asian, 4.3% Mixed, 0.4% Other	91.9% White, 2.5% Asian, 5.0% Mixed, 0.6% Other	89.5% White, 1.2% Black 8.1% Asian, 1.2% Other
Antibiotic use (% yes)	8.9%	13.7%	11.6%
Probiotic supplements (% yes)	12.3%	13.0%	11.6%
FAV *M* (SD)	73.10 (20.55)	71.26 (21.66)	67.24 (22.96)
Omega‐3 foods *M* (SD)	5.67 (4.03)	5.61 (3.74)	5.07 (3.20)
Probiotic foods *M* (SD)	20.66 (6.93)	20.47 (6.93)	19.31 (7.68)
Gastro symptoms *M* (SD)	0.39 (0.78)	1.00 (1.55)	1.88 (2.10)
Depression *M* (SD)	3.34 (3.29)	7.65 (3.80)	17.91 (3.87)

Abbreviations: CD, current depression; FAV, fruit and vegetables; HC, healthy controls; LD, lifetime depression; *M*, mean; SD, standard deviation.

### 2.2 Recruitment

All participants were recruited via Prolific, an online research participant recruitment platform. The group with depression was collected from a sample previously screened for self‐reported depression diagnoses by a project run within the Centre for Innovation in Mental Health at the University of Southampton. The healthy control group was recruited from the overall sample using a filter to screen for individuals without a self‐reported mental health problem.

### 2.3 Measures

#### 2.3.1 Dietary Intake

The Fruit And Vegetable VAriety index (FAVVA, Ashton et al. 2019) was used to measure the frequency and variety of fruit and vegetable (FAV) intake. The original rating scale was adjusted to reduce the frequency range from an 8‐ to a 7‐point category scale ranging from ‘Never’ to ‘2 or more times per day’ to align with other food intake questionnaires included in this study. Participants were asked to rate how often they had consumed 21 vegetable and 11 fruit items over the past month. FAVVA scores have shown moderate‐to‐strong correlations with carotenoids (Ashton et al. 2019), robust biomarkers of fruit and vegetable intake (Couillard et al. 2016). Scores could range from 0 to 192 with higher scores reflecting more frequent fruit and vegetable consumption.

The N‐3 PUFA Food Frequency Questionnaire (Sublette et al. 2011) was used to measure omega‐3 fatty acid foods' intake. The questionnaire estimates mean dietary intakes of omega‐3 fatty acids EPA and DHA and demonstrated significant correlations with their respective mean plasma levels (DHA Spearman's *r* = 0.50; *p* < 0.0001; EPA *r* = 0.38; *p* = 0.002). The measure consists of 21 items. Six items ask unique information about gender, time of last meal and consumption of fish or shellfish within the past 24 h and the past week, and type of fish/shellfish consumed. Six items assess the frequency and amount of a variety of seafood and fish, as well as walnuts, flaxseed, flaxseed oil, cod liver oil and canola oil consumed in the past 6 months. The original rating scale was adjusted to reduce the response scale from a 10‐ to a 7‐point category scale ranging from ‘Never’ to ‘2 or more times per day’ to align with other food intake questionnaires included in this study. Only the ratings for these six items were included for this study. The other items were follow‐up questions asking about portion sizes. For the current study, higher scores reflected higher consumption of omega‐3‐rich foods. Scores could range from 0 to 36.

The Prebiotic and Probiotic Food Frequency Questionnaire (Parhizgar et al. 2021) was used to measure the frequency of probiotic and prebiotic food consumption. The original measure included 16 items. Five items (fruits, onions, spinach, cabbage and probiotic capsules) were excluded in the current study as they duplicated items from other dietary intake measures. Two items (‘Salty products such as pickles, salted cabbage and salted olives’ and ‘Pickled products’) were merged into one ‘Salty/pickled products (eg, olives, pickled cucumber/gherkins, sauerkraut and kimchi)’ to adapt to the cuisine in the United Kingdom.

The original rating scale was adjusted from a 5‐ to a 7‐point scale ranging from ‘Never’ to ‘2 or more times per day’ to align with other food intake questionnaires included in this study. Participants were asked to rate how often they had consumed 10 pre‐ and probiotic food items over the past month. Scores in the current study could range from 0 to 60 with higher scores reflecting more frequent consumption of probiotic/prebiotic‐rich foods. The measure showed acceptable internal consistency with a Cronbach's α coefficient of 0.57 in the current study.

#### 2.3.2 Physical Activity

The General Practice Physical Activity Questionnaire (GPPAQ) was used to measure the level of physical activity. The 3‐item measure asked participants about the type and amount of physical activity involved in their work, the time spent doing different physical activities during the past week and their typical walking pace. The GPPAQ is a validated screening tool widely used to measure physical activity levels in adults (Ahmad et al. 2015). Physical activity scores measured by the GPPAQ were categorised into inactive, moderately inactive, moderately active and active groups following the measure's guidance.

#### 2.3.3 Gastrointestinal Health

The One‐page System Questionnaire (OSQ, Pereira et al. 2014) was used to measure the frequency of GI symptoms participants had experienced on the day, using a 4‐point categorical scale ranging from ‘Absent, I did not have this symptom at all’ to ‘Severe, I had this symptom often’. The current study used ‘subscale C’ which included a variety of common gastrointestinal symptoms: nausea, vomiting, heartburn, abdominal pain, diarrhoea and constipation. Two items ‘headache’ and ‘breathlessness’ were excluded as these were related to iron supplementation in the original study. Higher scores reflect a higher frequency of GI symptoms. Scores could range from 0 to 18. The OSQ was shown to be a valid measure as it includes symptoms associated with gut microbiota imbalance (Chong et al. 2019).

The Bristol stool chart (Lewis and Heaton 1997) asked participants to describe the shape and consistency of their most recent bowel movement on a 7‐point visual chart. It is a valid and reliable measure of bowel movement widely used in clinical and research settings (Blake et al. 2016). Stool consistency measured by the Bristol Stool Scale has demonstrated strong correlations with gut microbiota richness and composition (Vandeputte et al. 2016), which are widely considered as a proxy of gastrointestinal health (Lloyd‐Price et al. 2016). The measure includes a stool scale ranging from types 1 to 7 with the lowest numbers indicating constipation and the higher numbers suggesting diarrhoea. In regression analysis, the scores were converted to interval variables with score ‘4’ replaced with ‘0’ to represent the ‘healthy’ bowel movement, with other scores deviating from it (‘1, 2, 3, 4, 5, 6, 7’ converted to ‘3, 2, 1, 0, 1, 2, 3’).

#### 2.3.4 Depression

The 9‐item PHQ‐9 (Kroenke et al. 2001) was used to assess the presence and severity of depression. The measure asks to rate the frequency of depression symptoms over the past 2 weeks using a 4‐point scale ranging from ‘Not at all’ to ‘Nearly every day’ with higher scores reflecting higher rates of depressive symptoms. Total scores of 5, 10, 15 and 20 represent cut‐off points for mild, moderate, moderately severe and severe depression, respectively. To determine MDD diagnoses, an algorithm was followed based on the Diagnostic and Statistical Manual of Mental Disorders, retrieved from the original PHQ study (Spitzer et al. 1999). The algorithm method requires a total of at least five symptoms rated as at least 2 (more than half the days), except for the suicidal ideation item, which counts as one of the five symptoms if it is rated as 1 (several days) or above. PHQ‐9 demonstrated excellent internal reliability with a Cronbach's α of 0.91 in the current study.

### 2.4 Procedure

Participants were directed from Prolific to the web‐based online survey tool Qualtrics via a link. They were asked to provide demographic information including age, gender and ethnicity, and were asked about alcohol, antidepressant and supplement consumption. Participants then completed the self‐report measures on their dietary intake, physical activity, GI health and mental health.

### 2.5 Analysis

#### 2.5.1 Data Preparation

Individual scores were excluded from analysis if more than 10% of data points per measure were missing. This resulted in only a small amount of data points being excluded across variables (2–8 per variable). As a result of this strategy, total scores on individual outcome measures could not be calculated for *n* = 12 participants (GI health: *n* = 6, depression: *n* = 2, probiotic food intake: *n* = 2, FAV: *n* = 2). Data on the Bristol stool chart (single‐item measure) was missing for *n* = 8 participants. Apart from one participant (missing data on Bristol stool chart and probiotic food intake), no participant had missing total scores on more than one of the main outcome measures. Extreme outliers were identified by converting the participants' scores into z‐scores (> 3.29 or < −3.29) and subsequently winsorised (Tabachnick, Fidell, and Ullman 2013; winsorisation: GI problems: *n* = 9 total scores, omega‐3 intake: *n* = 4 total scores).

### 2.6 Statistical Analysis

Data were analysed using IBM SPSS Statistics 28.0. Bootstrapping of 5000 samples with 95% confidence intervals was applied due to skewness in the data. Demographic variables were compared across groups using analysis of variance (ANOVA) and Pearson chi‐square tests. Bonferroni post hoc tests were used for ANOVA multiple comparisons. Post hoc testing for chi‐square analysis was carried out after choosing the Bonferroni‐corrected *p*‐value for each contingency table. Subsequently, the *p*‐value was calculated for each cell, and only cells with a *p*‐value lower than the Bonferroni‐corrected *p*‐value were reported as significant (MacDonald and Gardner 2000). Pearson correlations with bootstrapped confidence intervals were run to test for associations between FAV, omega‐3 and probiotic foods, GI health and depression.

The potentially mediating role of GI health was assessed with the Hayes PROCESS macro (Hayes 2017) for SPSS using model 4, with bootstrapped (5000 bootstraps) 95% confidence intervals. Assumptions for regression analysis were met except for homoscedasticity. The HC3 method was therefore used to produce heteroscedasticity‐consistent standard error estimates (Long and Ervin 2000). FAV, omega‐3 and probiotic foods, and physical activity were defined as predictors, GI health (GI symptom scores and bowel movement) as the mediating variable and depression as the outcome variable. This resulted in eight simple mediation models being constructed and tested.

Given the cross‐sectional nature of the data, causal assumptions cannot be drawn from the results of the mediation analyses. Exploratory mediation analysis can however still be useful to identify potential mediators as targets for future studies (van Kesteren and Oberski 2019).

## 3 Results

### 3.1 Demographic and Clinical Characteristics

The demographic information and clinical characteristics are presented in Table 1. There were significant differences between HC, LD and CD groups in age (*F*[2478] = 4.152, *p* = 0.016). Post hoc analysis revealed that age was significantly higher in the HC group compared to the CD group (*p* = 0.015). There were no significant differences between the groups in sex (*χ* ^2^[2] = 0.559, *p* = 0.756), alcohol consumption (*F*[2470] = 1.420, *p* = −0.243), antibiotic use (*χ* ^2^[2] = 2.225, *p* = 0.329) or probiotic supplement intake (*χ* ^2^[2] = 0.108, *p* = 0.947).

### 3.2 Group Comparison for Dietary Intake, Physical Activity, Gastrointestinal Health and Depression

**FIGURE 1:**
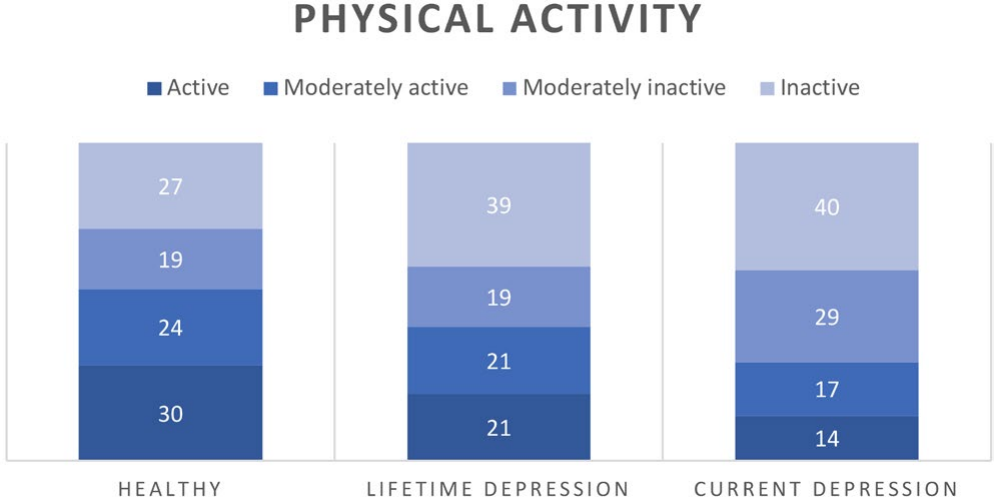
Percentages of participants categorised inactive, moderately inactive, moderately active and active groups.

There were no significant differences among the three groups in FAV (*F*[2477] = 2.370, *p* = 0.095), omega‐3 (*F*[2479] = 0.834, *p* = 0.435) and probiotic (*F*[2477] = 1.181, *p* = 0.380) food intake. Physical activity (Figure 1) differed significantly across the groups (*χ* ^2^[6] = 16.926, *p* = 0.010). Post hoc analysis revealed that the difference was explained by the higher percentage of HCs in the active group compared to the CD group (30% vs 14%, *p* = 0.05).

**FIGURE 2:**
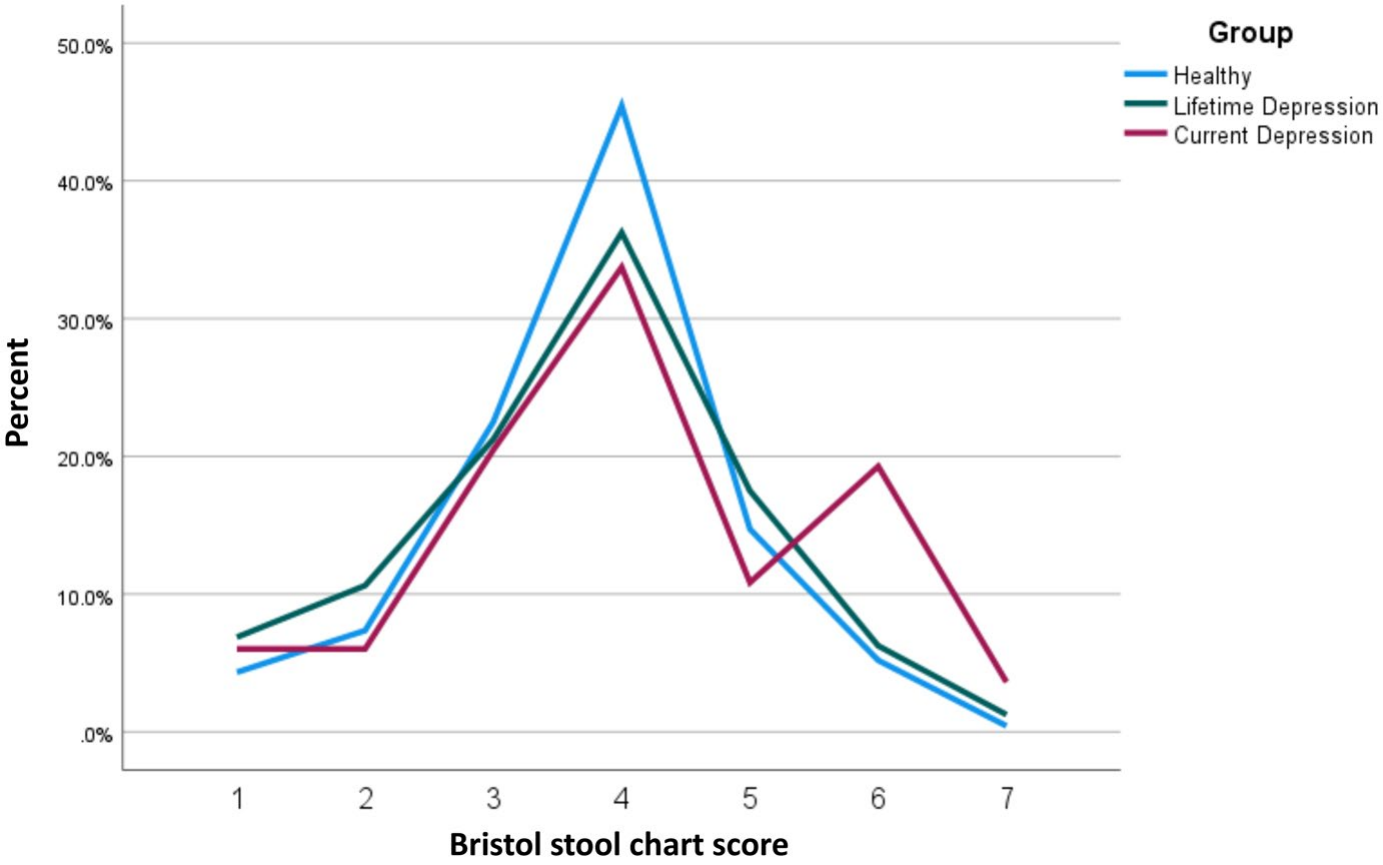
Bowel movement type categorisation by group.

The GI symptom scores were also significantly different between the three groups (*F*[2, 473] = 38.342, *p* < 0.001). Post hoc analysis showed that the GI symptom scores were significantly different between all pair comparisons, with highest scores in the CD group and lowest scores in the HC group (all *p* < 0.001). The bowel movement types significantly differed between groups (*χ* ^2^(12) = 28.76, *p* = 0.004). The HC group showed 46% type 4 bowel movements compared to 34% in the CD group, see Figure 2. However, the result for the difference in bowel movements did not survive Bonferroni‐correction for multiple testing (*p* < 0.004).

As expected, depression scores significantly differed between HC, LD and CD groups (*F*[2, 479] = 525.118, *p* < 0.001). Post hoc analysis revealed that the depression scores remained significant after Bonferroni‐correction between all pair comparisons within the three groups (all *p* < 0.001).

### 3.3 Correlations Between Variables

The bootstrapped bivariate correlations between the variables are shown in Table 2. FAV intake showed a significant negative correlation with GI symptoms (95% CI [−0.245, −0.070]) and depression scores (95% CI [−0.228, −0.052]). Omega‐3 intake demonstrated a significant negative correlation with GI symptom scores (95% CI [−0.201, −0.024]) and depression scores (95% CI [−0.200, −0.023]). There was no significant relationship between probiotic food intake and GI symptoms, or between probiotic food intake and depression scores. GI symptoms showed a statistically significant positive relationship with depression scores (95% CI [0.118, 0.290]).

**TABLE 2:** Pearson bivariate correlations with 95% confidence intervals (5000 bootstraps) among all study variables (*n* = 477).

	1. FAV	2. Omega‐3 foods	3. Probiotic foods	4. GI symptoms
1. FAV				
2. Omega‐3 foods	0.42** [0.343, 0.491]			
3. Probiotic foods	0.41** [0.333, 0.482]	0.44** [0.365, 0.510]		—
4. GI symptoms	−0.16** [−0.245, −0.070]	−0.12** [−0.201, −0.024]	−0.08 [−0.155, 0.024]	
5. Depression	−0.14** [−0.228, −0.052]	−0.11* [−0.200, −0.023]	−0.09* [−0.176, 0.002]	0.47** [0.392, 0.533]

*Note:* Bootstrapped point estimates are for *k* = 5000 samples.

Abbreviations: FAV, fruit and vegetable; GI, gastrointestinal.

* Significant at the 0.01 level.

** Significant at the 0.05 level.

### 3.4 Exploratory Mediation Analyses

Based on correlational patterns, GI symptoms were explored as potential mediators of the association between FAV and omega‐3 intake with depression.

#### 3.4.1 GI Symptoms as the Mediator

##### 3.4.1.1 FAV

The overall model was significant, *R* ^2^ = 0.14, *F*(2, 471) = 44.00, *p* < 0.001, accounting for 14% of the variance in predicting depression group. As shown in Table 3, the effect of FAV intake on depression group membership was statistically mediated by GI symptoms (*β* = −0.0020, 95% CI [−0.0032, −0.0008]) with no direct effect of FAV intake on the depression group (*β* = −0.0013, 95% CI [−0.0045, 0.0016]).

**TABLE 3:** Results for bootstrapped estimates of indirect and direct effects of the mediation model.

Mediator	Predictor	*N*	*R* ^2^	Indirect effect *β* (bootstrap 95% CI)	Direct effect *β* (bootstrap 95% CI)
GI symptoms	FAV intake	474	0.14	−0.0020 (**−0.0032, −0.0008**)	−0.0013 (−0.0045, 0.0016)
	Omega‐3 intake	476	0.14	−0.0082 (**−0.0143, −0.0020**)	−0.0021 (−0.0179, 0.0142)
	Probiotic foods	474	0.14	−0.0024 (−0.0063, 0.0016)	−0.0047 (−0.0141, 0.0045)
	Physical activity	476	0.15	−0.1091 (**−0.1723, −0.0546**)	−0.2212 **(−0.3885, −0.0556)**
Bowel movement	FAV intake	472	0.03	−0.0006 (**−0.0013, −0.0001**)	−0.0030 (−0.0063, 0.0001)
	Omega‐3 intake	474	0.02	−0.0019 (−0.0053, 0.0006)	−0.0082 (−0.0253, 0.0090)
	Probiotic intake	473	0.02	−0.0007 (−0.0024, 0.0008)	−0.0053 (−0.0153, 0.0043)
	Physical activity	474	0.05	−0.0203 (−0.0514, 0.0031)	−0.2919 (**−0.4627, −0.1233**)

*Note:* Bootstrapped point estimates are for *k* = 5000 samples. Significant differences between the groups are in bold values.

Abbreviations: FAV, fruit and vegetable; GI, gastrointestinal.

##### 3.4.1.2 Omega‐3

The overall model was significant, *R* ^2^ = 0.14, *F*(2, 473) = 44.24, *p* < 0.001, accounting for 14% of the variance in predicting depression group. The effect of omega‐3 intake on depression group membership was statistically mediated by GI symptoms (*β* = −0.0082, 95% CI [−0.0143, −0.0020]) with no direct effect of omega‐3 intake on the depression group (*β* = −0.0021, 95% CI [−0.0179, 0.0142]).

##### 3.4.1.3 Physical Activity

The overall model was significant, *R* ^2^ = 0.15, *F*(4, 471) = 24.95, *p* < 0.001, accounting for 15% of the variance in predicting depression group. There was a significant direct effect of physical activity on the depression group (*β* = −0.2212, 95% CI [−0.3885, −0.0556]). Additionally, the effect of physical activity on depression group membership was statistically mediated via GI symptoms (*β* = −0.1091, 95% CI [−0.1723, −0.0546]).

#### 3.4.2 Bowel Movement as the Mediator

##### 3.4.2.1 FAV

The overall model was significant, *R* ^2^ = 0.03, *F*(2, 469) = 6.89, *p* = 0.001, accounting for 3% of the variance in predicting depression group. As shown in Table 3, the indirect effect of FAV intake on the likelihood of being in the depression group was found to be statistically significant (*β* = −0.0006, 95% CI [−0.0013, −0.0001]). The direct effect of FAV intake on the depression group via bowel movement was not significant (*β* = −0.0030, 95% CI [−0.0063, 0.0001]).

##### 3.4.2.2 Omega‐3

The overall model was significant, *R* ^2^ = 0.02, *F*(2, 473) = 44.24, *p* = 0.006, accounting for 2% of the variance in predicting depression group. The indirect and direct effects of omega‐3 intake on the depression group were not significant.

##### 3.4.2.3 Physical Activity

The overall model was significant, *R* ^2^ = 0.05, *F*(4, 469) = 6.65, *p* < 0.001, accounting for 5% of the variance in predicting depression group. The indirect effect of physical activity on the likelihood of being in the depression group was not significant; however, the direct effect was significant (*β* = −0.2919, 95% CI [−0.4627, −0.1233]).

## 4 Discussion

The current study examined the differences in dietary intake, PA and GI health between healthy controls, lifetime depression and current depression groups. The GI symptom scores were significantly different between the three groups where HC showed the lowest and CD the highest frequency of GI symptoms. Additionally, we found a moderate correlation between GI and depressive symptoms. These results are in line with previous evidence demonstrating differences in the gut microbiota in individuals with depression when compared to healthy populations (Knudsen et al. 2021) and add to the evidence base by suggesting there are also differences in the microbial compositions between lifetime and current depression populations. Elevation of GI symptoms in groups with current depression and a lifetime diagnosis is consistent with theoretical models that highlight ‘leaky‐gut syndrome’ as a potential mechanism linking gut health and depression (Kouba et al. 2024). It is of particular interest that, relative to never‐depressed controls, GI symptoms were elevated in participants with a lifetime diagnosis of depression, but no current depressive episode. This may reflect increased permeability of the intestinal barrier as a latent risk factor for recurrent depression. As our data are correlational, we cannot rule out the possibility of GI symptoms developing as a result of depression. For instance, changes in eating behaviour or weight can be a symptom of depression (American Psychiatric Association 2013).

Physical activity levels were also significantly different between the groups, with HC having a significantly higher percentage of individuals categorised as ‘active’, followed by LD and CD groups (30%, 21% and 14%, respectively). This is in line with previous research findings summarised by an umbrella review. Results across randomised controlled trials of exercise interventions supported a positive effect of exercise on depressive symptoms (Singh et al. 2023). In addition to a direct effect of PA on depression group membership, exploratory mediation analysis identified GI health as a potential mediator between PA and depression.

Contrary to what was expected based on previous research (Liu et al. 2016; Yang et al. 2018), no significant differences were found in FAV, omega‐3 and probiotic foods consumption between the three groups. The small sample size in the CD group could partly explain why the difference could not be detected. Furthermore, previous studies have compared healthy populations with individuals with depression. It is possible that grouping the participants by healthy, lifetime and current depression did not allow the detection of a significant difference in the consumption of these foods. Correlational analysis also showed no significant correlation between probiotic food consumption and depressive or GI symptoms. In line with our assumptions, both depressive symptoms and GI symptoms correlated negatively with FAV and omega‐3 consumption. The strength of the effect was however very weak. Exploratory mediation analyses suggested that GI symptoms partially mediated the relationship between FAV intake and depression and omega‐3 intake and depression. Bowel movements also partially mediated the relationship between FAV intake and depression. These data indicate that the effects of FAV intake on depression partly work through the GI system (ie, GI symptoms and bowel movements). The same was suggested for the effects of omega‐3 intake, but only via GI symptoms and not bowel movements. These findings help to further understand the potential mechanisms between dietary habits and depression. The relatively weak effects, however, suggest that other factors related to GI health may be more important. One of these factors may be the consumption of inflammation‐promoting foods (Wang et al. 2019).

GI health did not correlate with probiotic food intake and depression. These results may be explained by the difficulties with measuring probiotic food intake. There is an issue with the authenticity of probiotic food products not being compliant with their labels. A recent review concluded that it was common that probiotic foods such as yoghurts did not include the microbial species advertised on labels or contained significantly smaller counts than promised. The same issue was found for probiotic supplements (Fusco et al. 2022). Therefore, it is possible that the self‐reports did not reflect an accurate intake of probiotics. In addition to the authenticity issue of probiotic foods, the internal consistency of the probiotic food measure in the current data showed only an acceptable level of Cronbach's alpha. Thus, it is possible that the measure did not effectively determine the participants' consumption of probiotic foods. The original measure was validated in an Iranian population (Parhizgar et al. 2021), and despite the attempts to adapt the questionnaire to the cuisine in the United Kingdom, the results suggested that it may have not been a reliable measure in this study population.

Our findings suggest that consumption of FAVs and food rich in omega‐3 may play a very small, but protective role in depression. A meta‐analysis of 27 observational studies on the association between fruit and vegetable intake and depression, however, reported a risk‐lowering effect of between 14% and 25%. Moreover, each 100 g increase in fruit or vegetable consumption was associated with a 3% lower depression risk. Results, however, indicated significant heterogeneity with several studies finding no significant effect (Saghafian et al. 2018). A study by Scheelbeek et al. (2020) showed that the consumption of these foods is often low, suggesting that only 26% of the UK population consumes recommended amounts of FAV. This highlights the need to deliver better education for the public and healthcare professionals about the role of diet and PA in sustaining mental health. Diet and PA are cost‐effective methods to support mental health and should be further promoted by healthcare professionals. Our results particularly support a potentially protective role of PA. There is scope for clinical implications establishing therapies that promote a healthy diet and PA for the prevention and complementary treatment for depression, and maintenance of mental health. It also has potential to offer a method of support for people with depression who are hesitant about antidepressants. In terms of psychological therapies, the findings highlight the importance of focusing on the physical activity component, such as the behavioural activation technique in Cognitive Behavioural Therapy as compared to sedentary activities.

The results also indicated that diet and particularly PA might influence psychopathology through the effects on GI health, possibly via exercise‐induced adaptation of the microbiome and anti‐inflammatory effects (Sohail et al. 2019). This helps us further understand the mechanisms for the protective effects dietary intake and PA have for depression. These findings were also an important addition to the evidence base suggesting there are differences in GI microbial compositions between individuals who have experienced a depressive episode in the past and who are currently suffering from depression.

These data should however be interpreted considering a number of limitations. The data are cross‐sectional and correlational and therefore cannot provide conclusive information about causal relationships, and further prospective cohort studies are needed. Furthermore, self‐report measures can give an estimate of an individual's dietary intake but have limitations on the accuracy of nutrient intake. This may be particularly problematic with probiotic foods where microbial species advertised have been found noncompliant with their labels (Fusco et al. 2022). Therefore, future studies could examine biomarkers for more objective measures of nutrient intake.

While our study examined fruit and vegetable consumption, it did not assess consumption of wholegrain foods or look at fibre intake specifically. Given their positive impact on gut microbiome composition (Ye et al. 2022), future studies should assess and analyse fibre and/or wholegrain food intake. It is further important to highlight that the current study only measured dietary intake that is considered protective for depression and did not measure risk food groups such as processed foods and sugary food intakes which promote inflammation (Tristan Asensi et al. 2023). Future studies could include both to investigate their interplay in the context of depression. Also, the current study population was largely reported from White ethnic backgrounds and therefore limits the generalisability to people from minority ethnic groups. More studies including a variety of ethnic backgrounds are needed to better understand the association between lifestyle factors, GI health and mental health.

To our knowledge, this was the first study to compare the differences in lifestyle factors and GI health between lifetime depression and current depressive episode. The findings highlight the potential involvement of the gastrointestinal system and the potential importance of considering lifestyle interventions in the prevention of depression and maintenance of mental health. It also added to our understanding of potential mechanisms linking these lifestyle factors to depression aetiology which can inform future longitudinal or experimental studies.

## Conflicts of Interest

The authors declare no conflicts of interest.

## Data Availability

Data are available on request to bona fide researchers only after the embargo period is lifted on 31/12/24. Data are available in EPrints (http://eprints.soton.ac.uk/id/eprint/491156) at https://doi.org/10.5258/SOTON/D3075.
